# The uremic solute 3-carboxy-4-methyl-5-propyl-2-furanpropionate (CMPF) may enhance eryptosis and increase erythrocyte osmotic fragility through potential activation of PIEZO1

**DOI:** 10.1093/ndt/gfae275

**Published:** 2024-11-20

**Authors:** Beatriz Akemi Kondo Van Spitzenbergen, Gabriela Bohnen Andrade, Erika Sousa Dias, Júlia Bacarin Monte Alegre, Gabriela Ferreira Dias, Nadja Grobe, Andrea Novais Moreno-Amaral, Peter Kotanko

**Affiliations:** Department of Postgraduate Program in Health Sciences, Research Laboratory of Anemia and Immunology (LabAIRe), Pontifícia Universidade Católica do Paraná, Curitiba, PR, Brazil; Department of Postgraduate Program in Health Sciences, Research Laboratory of Anemia and Immunology (LabAIRe), Pontifícia Universidade Católica do Paraná, Curitiba, PR, Brazil; Department of Postgraduate Program in Health Sciences, Research Laboratory of Anemia and Immunology (LabAIRe), Pontifícia Universidade Católica do Paraná, Curitiba, PR, Brazil; Department of Postgraduate Program in Health Sciences, Research Laboratory of Anemia and Immunology (LabAIRe), Pontifícia Universidade Católica do Paraná, Curitiba, PR, Brazil; Renal Research Institute, New York, NY, USA; Renal Research Institute, New York, NY, USA; Department of Postgraduate Program in Health Sciences, Research Laboratory of Anemia and Immunology (LabAIRe), Pontifícia Universidade Católica do Paraná, Curitiba, PR, Brazil; Renal Research Institute, New York, NY, USA; Department of Nephrology, Icahn School of Medicine at Mount Sinai, New York, NY, USA

**Keywords:** eryptosis, osmotic fragility, PIEZO1, renal anemia, uremic toxin

## Abstract

**Background and hypothesis:**

In patients with advanced CKD the lifespan of red blood cells (RBCs) is often shortened, a condition attributed to the ‘uremic milieu.’ We reported recently that the uremic solute 3-carboxy-4-methyl-5-propyl-2-furanpropionate (CMPF) shares structural similarities with Jedi1, a chemical activator of the mechanosensitive cation channel PIEZO1, whose activation increases calcium influx into cells. Against this backdrop, we hypothesized that CMPF may induce premature RBC death (eryptosis) through prolonged CMPF-induced activation of PIEZO1 located on RBCs. To test this hypothesis, we explored if CMPF, at concentrations found in uremia, interacts with PIEZO1 located on RBCs, increases intracellular calcium (icCa^2+^), and induces eryptosis.

**Methods:**

RBCs from healthy individuals were incubated with CMPF or Jedi1 (both at a concentration of 87 µM), in the presence or absence of the PIEZO1 inhibitor GsMTx-4 (2 µM). We challenged RBCs osmotically through incubation in solutions of NaCl at concentrations between 3.0 and 9.0 g/L and determined their osmotic fragility. Using flow cytometry, we quantified in incubated RBCs icCa^2+^ levels and phosphatidylserine exposure, a cellular marker of eryptosis.

**Results:**

Incubation of RBCs with CMPF and Jedi1 significantly increased RBC osmotic fragility, an effect prevented by GsMTx-4. At 6.0 g/L NaCl, incubation with CMPF and Jedi1 increased exposure of phosphatidylserine and elevated icCa^2+^ levels of RBCs, indicating increased eryptosis. Notably, at an isotonic NaCl concentration of 9.0 g/L, CMPF—but not Jedi1—significantly increased RBC phosphatidylserine exposure and icCa^2+^ levels; both effects were diminished by GsMTx-4.

**Conclusion:**

Our findings support the hypothesis that CMPF may function as an endogenous activator of PIEZO1, increase icCa^2+^ levels, trigger eryptosis, and, through this pathway, possibly shorten the RBC lifespan. To what extent these *in vitro* findings are operative in advanced CKD warrants clinical studies.

## Graphical Abstract

**Graphical Abstract:**
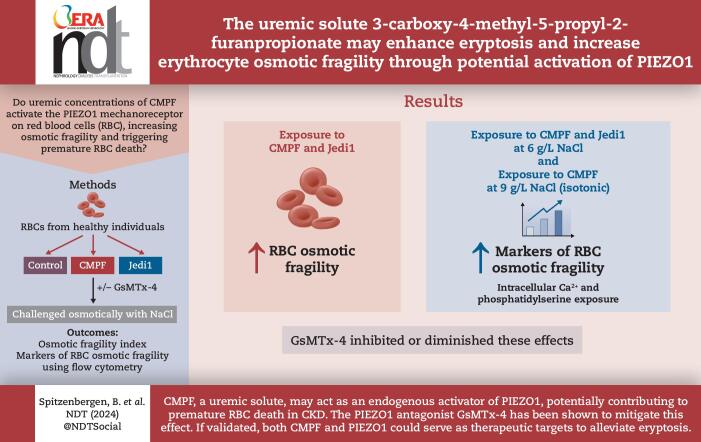

KEY LEARNING POINTS **What was known:**

- In patients with CKD, the lifespan of red blood cells (RBCs) is often shortened, a condition attributed to the ‘uremic milieu’. It has been hypothesized that the uremic solute 3-carboxy-4-methyl-5-propyl-2-furanpropionate (CMPF) induces premature RBC death (eryptosis) by activating the mechanosensitive cation channel PIEZO1 located on RBCs.

**This study adds:**

- Incubation of RBCs with CMPF at uremic concentrations increased eryptosis, intracellular calcium, and RBC osmotic fragility. These effects were blocked by the PIEZO1 inhibitor GsMTx-4. The findings support the hypothesis that CMPF may function as an endogenous activator of RBC PIEZO1 and, through this pathway, possibly shorten RBC lifespan.

**Potential impact:**

- So far, the molecular mechanisms of toxicity have only been clarified for a few uremic solutes. If confirmed by other groups and complementary methods (e.g. patch clamping), our findings may open new therapeutic approaches to mitigate or even prevent premature death of RBCs in patients with advanced CKD.

## INTRODUCTION

The etiology of renal anemia is multifactorial and includes inappropriately low erythropoietin (EPO) production, absolute and functional iron deficiency, and shortened red blood cell (RBC) lifespan. Premature RBC death (eryptosis) plays a pivotal role in the pathogenesis of renal anemia [1, 2]. In patients with chronic kidney disease, eryptosis rates are increased compared with healthy individuals [3]. Augmented eryptosis is attributed to the ‘uremic milieu’, an ill-defined condition characterized by increased uremic retention solutes [4, 5], and osmotic [6] and oxidative stress [7]. On a molecular level, Ca^2+^ influx through selective cation channels instigates the translocation of phosphatidylserine (PS) to the outer leaflet of the RBC plasma membrane. This exposure of PS serves as a signal for eryptosis [8] and facilitates recognition, phagocytosis, and degradation of RBCs by macrophages [1] and proinflammatory monocytes [9].

In humans, RBCs traverse the capillary bed ∼100 000–200 000 times during their 120-day lifespan. The passage of RBCs through narrow anatomical structures requires very fast changes in cell shape and volume [10]. The process is started by the mechanical stimulation of the mechanosensitive cation channel PIEZO1 located on the RBC plasma membrane. A conformational change of PIEZO1 increases its conductivity for Ca^2+^, Ca^2+^ influx, and a rise of intracellular Ca^2+^ (icCa^2+^). Elevated icCa^2+^ stimulates calcium-activated potassium KCa3.1 Gardos channels [11], resulting in RBC membrane hyperpolarization and shrinkage of RBCs due to a loss of K^+^, Cl^−^, and water. Furthermore, the calcium–calmodulin complex destabilizes the actin–adducin–band 4.1 complex, increasing the flexibility of the cross-linked spectrin network [12]. These intricate processes take only a few milliseconds and enable RBC passage through capillaries and other narrow anatomical structures. After completion of the passage, the mechanical stimulation of PIEZO1 ceases, the Ca^2+^ATPase restores icCa^2+^ levels to pre-passage levels, the Gardos channels close [13], and RBCs return to their pre-passage size.

While mechanical stress is the sole known physiological PIEZO1 activator, four synthetic compounds—Yoda1, Yoda2, Jedi1, and Jedi2—have been identified as chemical PIEZO1 activators [14–16]. These compounds increase in a dose-dependent manner the open probability of PIEZO1 without mechanical stimulation [16]. Jedi1 and Jedi2 bind to the extracellular domain at residues L15-16 and L19-20 of PIEZO1 [16], while Yoda1 binds to residues located in a hydrophobic region between transmembrane domains of the blade [14]. The Yoda2 binding sites are yet to be determined [15]. GsMTx-4, a peptide from spider venom [17], selectively inhibits cation-permeable channels, including PIEZO1 [17, 18].

**Figure 1:**
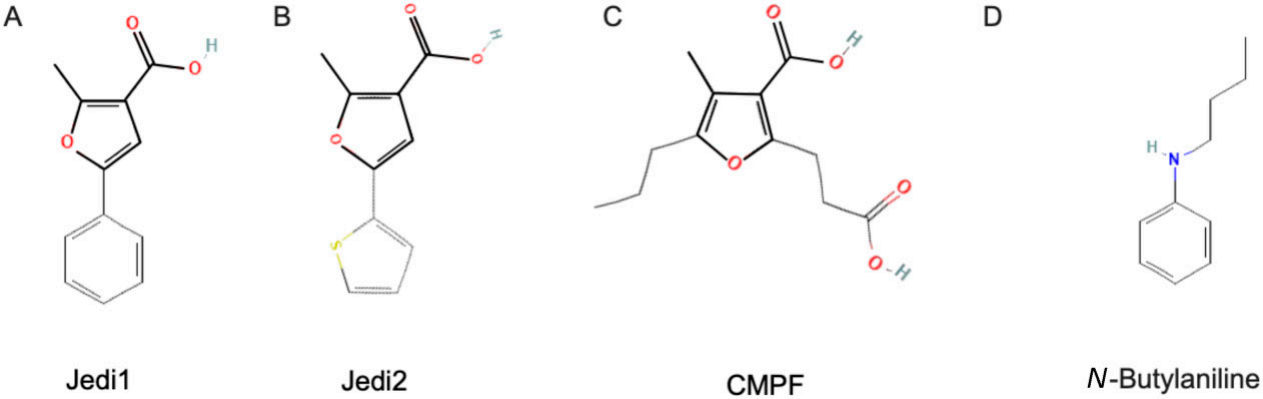
2D structures of Jedi1, Jedi2, CMPF, and *N*-butylaniline. (**A**) Jedi1 (2-methyl-5-phenylfuran-3-carboxylic acid), a chemical activator of PIEZO1. (**B**) Jedi2 (2-methyl-5-(thien-2-yl)-3-furoic acid), a chemical activator of PIEZO1. (**C**) CMPF (3-carboxy-4-methyl-5-propyl-2-furanpropionic acid), a protein-bound uremic retention solute derived from furan fatty acid metabolism. CMPF has been hypothesized to activate PIEZO1 like Jedi1 and Jedi2 due to their shared 3-carboxylic acid methyl furan moiety, the putative PIEZO1-activating domain; this structure is depicted in bold in (A), (B), and (C). (**D**) *N*-Butylaniline. This molecule lacks the 3-carboxylic acid methyl furan moiety and does not activate PIEZO1. Jedi1 CID #736 516; Jedi2 CID #2 796 026; CMPF CID #123 979; N-butylaniline CID #14310. Source of the compound's structure: PubChem, accessed on 30 August 2024.

Recently, we discovered that Jedi1 and Jedi2 share structural similarities with the uremic solute 3-carboxy-4-methyl-5-propyl-2-furanpropionate (CMPF) [19] (Fig. 1). CMPF is a metabolite of furan fatty acid metabolism. In CKD patients treated with dialysis, CMPF levels are increased 5- to 15-fold compared with healthy individuals [20]. CMPF is highly protein-bound (>95%) and hence poorly cleared by hemodialysis [20, 21]. Its uremic toxicity is ill-defined, and one study suggested that CMPF decreases erythroid colony formation *in vitro* [22]. Given the structural resemblance between CMPF and Jedi1 and Jedi2, we hypothesized that CMPF might activate PIEZO1 over an extended period of time and, in doing so, trigger Ca^2+^-dependent pathways that eventually result in eryptosis and shortened RBC lifespan [19].

Our research aimed to explore if CMPF, at concentrations found in uremia, interacts with PIEZO1 located on RBCs, increases icCa^2+^ and induces eryptosis. To that end, we conducted RBC incubation experiments with CMPF, Jedi1, and the PIEZO1 inhibitor GsMTx-4.

## MATERIALS AND METHODS

### Subjects

We recruited healthy subjects without a history, signs, or symptoms suggestive of kidney or inflammatory diseases. Exclusion criteria were the use of anti-inflammatory drugs or a history of blood transfusions. This study was approved by the ethics committee of Pontifícia Universidade Católica do Paraná (registration number 5.697.460). All study participants gave written informed consent before venipuncture.

### Isolation of red blood cells

We collected 4 mL of venous blood in 3.2% sodium citrate tubes (BD® Vacutainer, BD Biosciences, NJ, USA). For RBC isolation, whole blood was centrifuged (3000 × rpm, 15 min, 4°C). Plasma and buffy coat were discarded, and cells were washed twice with cold phosphate-buffered saline (PBS).

### Chemicals and solutions

CMPF and Jedi1 (Sigma–Aldrich, MO, USA) were reconstituted in DMSO at a concentration of 69.6 mM. GsMTx-4 (Abcam, Cambridge, UK) was reconstituted in PBS to a concentration of 244 µM. In end-stage kidney disease patients, the blood levels of total CMPF vary between 45 and 171 µM [23–27] with an average of 87 µM. Therefore, we used CMPF at a final concentration of 87 µM in the incubation experiments. To ascertain comparability, CMPF and Jedi1 were used at the same molar concentration.

### Red blood cell osmotic fragility testing

Isolated RBCs (200 µL) were suspended in 800 µL of PBS (pH 7.2) containing 4% of human serum albumin (HSA) to attain a hematocrit of 20%. This suspension was then incubated with or without GsMTx-4 (2 µM) [13] at room temperature. After 5 min, 1.25 µL of CMPF or Jedi1 stock solutions was added, resulting in a final concentration of 0.12% DMSO, and 87 µM of CMPF or Jedi1. Negative control consisted of RBC suspension incubated in 0.12% DMSO. Ten microliters of the resulting RBC suspension were transferred to either 1 mL of double-distilled water to induce complete hemolysis or increasing concentrations of NaCl solutions (3.0–7.0 g/L in 0.5 g/L increments, and 8.0 and 9.0 g/L). Double-distilled water and NaCl dilutions contained CMPF or Jedi1 (both 87 µM in 0.12% DMSO) or 0.12% DMSO (negative control). After 5 min of incubation at room temperature, the tubes containing RBCs were centrifuged at 1500 × rpm for 10 min, and 250 µL supernatant was subjected to hemoglobin optical density (OD) determination at 540 nm (Molecular Devices, CA, USA). The mean of duplicates was used to calculate the percentage hemolysis per NaCl concentration as follows:

\begin{equation*}{\mathrm{Hemolysis}}\left( \% \right) = 100 \times \left( {{\mathrm{ODs}}-{\mathrm{O}}{{{\mathrm{D}}}_0}} \right)/\left( {{\mathrm{O}}{{{\mathrm{D}}}_{\mathrm{t}}}-{\mathrm{O}}{{{\mathrm{D}}}_0}} \right)\end{equation*}

where OD_s_ is the optical density of the supernatant from RBCs incubated in saline solutions, OD_0_ is the optical density of the supernatant obtained from negative control RBCs incubated in isotonic saline, and OD_t_ is the optical density of the supernatant from RBCs incubated in double-distilled water [28]. The osmotic fragility index (OFI) was defined as the NaCl concentration that exerted 50% hemolysis.

### Eryptosis assays

The assessment of PS exposure via annexin-V binding was performed utilizing RBC pellets obtained from saline solutions used in the experiments with NaCl concentrations of 6.0 and 9.0 g/L. The RBC pellets were incubated in the dark for 15 min with annexin-V conjugated with allophycocyanin (APC) (ImmunoTools GmbH, Friesoythe, Germany) per manufacturer's instructions. The annexin-V incubation was followed by a PBS washing step and suspension in 4% paraformaldehyde in PBS (FixFACS). APC fluorescence was quantitated by flow cytometry using the FL4-A filter (Calibur BD Bioscience, Sparks, MD, USA) and expressed as mean fluorescence intensity (MFI).

Similarly, icCa^2+^ levels were measured by labeling RBC pellets with Fluo-4AM (Thermo Fisher Scientific, Waltham, MA, USA). Per manufacturer's instructions, RBCs were incubated for 40 min at 37°C in the dark. After labeling, the RBCs were washed with PBS and resuspended in FixFACS. Fluo-4AM fluorescence was measured by flow cytometry using the FL1-A filter, and the results are presented as MFI.

Forward scatter (FSC) and side scatter (SSC) were used to exclude debris from the analysis (Supplementary Data Fig. S1A). The cutoff for fluorescence positivity (dashed line in Supplementary Data Fig. S1B and C) was established based on the autofluorescence of cells incubated with saline solutions at 6.0 or 9.0 g/L NaCl without adding CMPF or Jedi1. This cutoff was determined for both fluorescence channels used in the analyses (LF4-A, Supplementary Data Fig. S1B, and LFA1-A, Supplementary Data Fig. S1C). This preliminary analysis served as a reference for the MFI to PS (Supplementary Data Fig. S2A) and to icCa^2+^ (Supplementary Data Fig. S2B). Since the DMSO vehicle did not affect FSC/SSC or autofluorescence, it was used as a negative control.

### Statistical analysis

Osmotic fragility curves (OFCs) were fitted from NaCl concentration versus % hemolysis data using the drc and ggplot2 packages (R statistical software version 4.1.1, R Foundation for Statistical Computing, Vienna, Austria). The OFI was computed from OFC fits.

The normal distribution of OFI was confirmed by the Kolmogorov–Smirnov test. Control and incubation OFIs were compared by paired *t*-test (GraphPad Prism version 9).

The percentage of RBCs displaying positive staining for PS exposure and icCa^2+^ was determined using FlowJo™ version 10.8 software (BD Life Sciences, Ashland, USA). MFI from each staining was analyzed by ANOVA and *P*-values were adjusted for multiple testing using the Holm–Šídák procedure (GraphPad Prism version 10.0.0 for Windows, Boston, MA, USA). A two-sided *P*-value < 0.05 was considered statistically significant.

## RESULTS

### Study subjects

RBCs were obtained from five healthy subjects. Their demographic characteristics and laboratory data are shown in Table 1. Average eGFR was 96 mL/min/1.73 m² (77.9–106.7 mL/min/1.73 m²).

**Table 1:** Demographic and laboratory characteristics of the blood donors.

**Parameter**	**Healthy subjects (*n* = 5)**
Age (years)	24 (23–38)
Sex	3 females; 2 males
Caucasians	5
BMI (kg/m^2^)	22.1 (20–27)
Hemoglobin (g/dL)	13 (12.6–16.7)
Serum creatinine (mg/dL)	0.8 (0.7–1.1)
Serum urea (mg/dL)	27 (23–35)
Serum albumin (g/dL)	4.2 (3.9–4.8)
eGFR (mL/min/1.73 m²)	96 (77.9–106.7)

Data are expressed as median (range). The eGFR was calculated using the CKD-EPI equation [39]. BMI: body mass index.

### Red blood cell osmotic fragility testing

**Figure 2:**
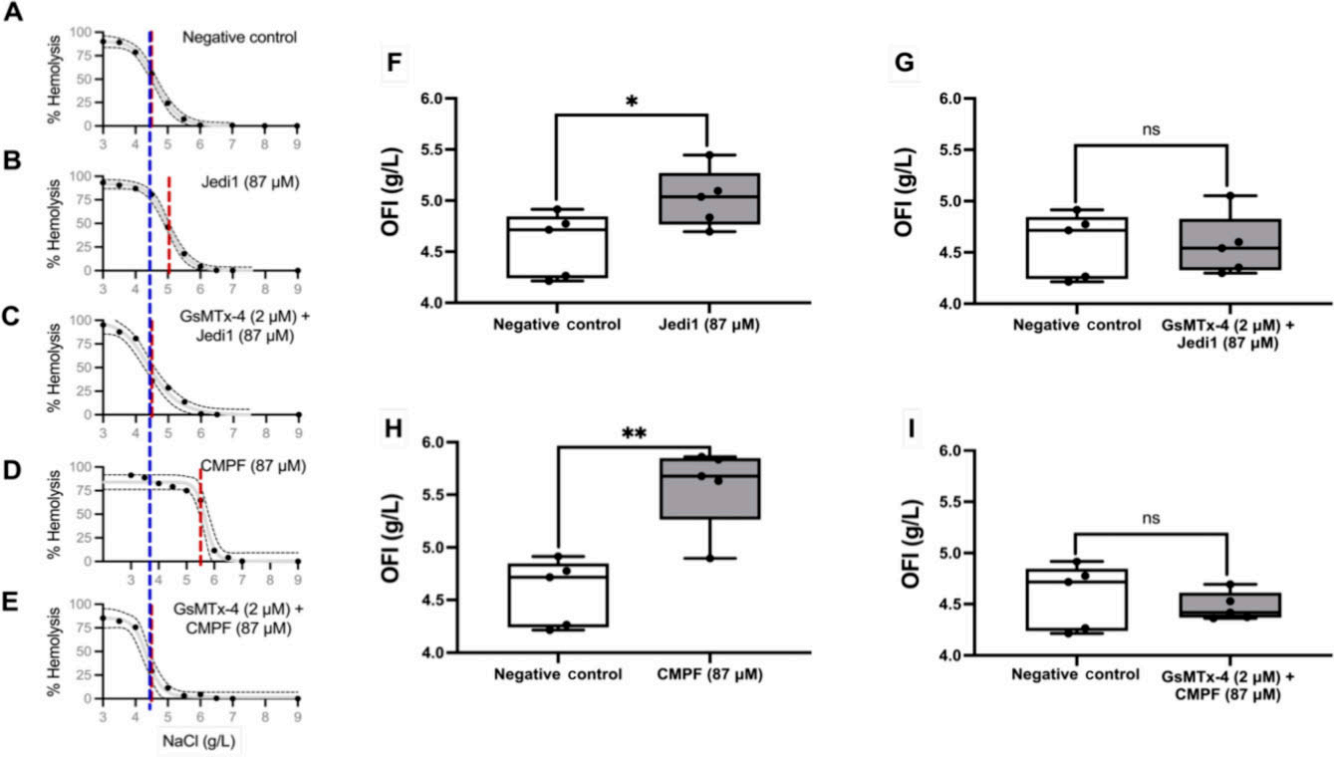
OFCs of isolated RBCs exposed to saline solutions ranging from 3 to 9 g/L and comparison of OFI across different experimental conditions. (**A**–**E**) RBC OFCs of the five healthy study subjects. (**A**) Negative control (PBS with 4% HSA and 0.12% DMSO). (**B**) RBCs incubated with Jedi1 (87 µM). (**C**) RBCs incubated with Jedi1 (87 µM) + GsMTx-4 (2 µM). (**D**) RBCs incubated with CMPF (87 µM). (**E**) RBCs incubated with CMPF (87 µM) + GsMTx-4 (2 µM). The red dashed line represents the OFI for each experimental condition, while the blue dashed line consistently represents the OFI of the negative control across all graphs. (**F**–**I**) OFI, expressed as the concentration of NaCl (g/L) required to achieve 50% hemolysis, is compared across different experimental conditions. The experimental conditions are indicated on the *x*-axis, with the negative control consisting of PBS with 4% HSA and 0.12% DMSO. (**F**) Comparison of OFI between Jedi1 (87 µM) and the negative control. (**G**) Comparison of OFI between GsMTx-4 (2 µM) + Jedi1 (87 µM) and the negative control. (**H**) Comparison of OFI between CMPF (87 µM) and the negative control. (**I**) Comparison of OFI between GsMTx-4 (2 µM) + CMPF (87 µM) and the negative control. *P < 0.05; **P < 0.005; ns, non-significant.

OFI results from all donors are shown in Fig. 2. Aggregated results are shown in Table 2. The negative control OFI was 4.57 ± 0.31 g/dL (Fig. 2A). Incubation with Jedi1 shifted the OFC to the right (Fig. 2B) and resulted in a significantly higher OFI (Fig. 2F; 5.02 ± 0.28 g/L; *P *= 0.031); pretreatment with GsMTx-4 prevented the effect of Jedi1 on OFI (Fig. 2C and G; 4.56 ± 0.29 g/L; *P *= 0.944). Incubation with CMPF resulted in a marked right shift of the OFC and a significant increase of OFI (Fig. 2D and H; 5.57 ± 0.39 g/L; *P *= 0.002); pretreatment with GsMTx-4 prevented that effect (Fig. 2E and I; 4.47 ± 0.13 g/L; *P *= 0.546).

**Table 2:** OFI of RBCs under different experimental conditions.

**Experimental condition**	**OFI (95% CI)**	**Mean difference from control (95% CI)**	***P*-value**
Negative control	4.57 ± 0.31 (4.18–4.97)	n/a	n/a
Jedi1 (87 μM)	5.02 ± 0.28 (4.66–5.37)	0.45 (0.06–0.83)	0.031
GsMTx-4 (2 μM) + Jedi1 (87 μM)	4.56 ± 0.29 (4.19–4.93)	−0.01 (−0.38 to 0.36)	0.944
CMPF (87 μM)	5.57 ± 0.39 (5.09–6.06)	1.00 (0.61–1.38)	0.002
GsMTx-4 (2 μM) + CMPF (87 μM)	4.47 ± 0.13 (4.30–4.64)	−0.10 (−0.52 to 0.32)	0.546

OFI is the NaCl concentration that exerts 50% hemolysis.

Negative control: PBS containing 0.12% DMSO and 4% HSA.

Data are presented as mean ± SD (95% confidence interval).

*P*-values were calculated using the paired *t*-test.

### Eryptosis and intracellular calcium analysis

**Figure 3:**
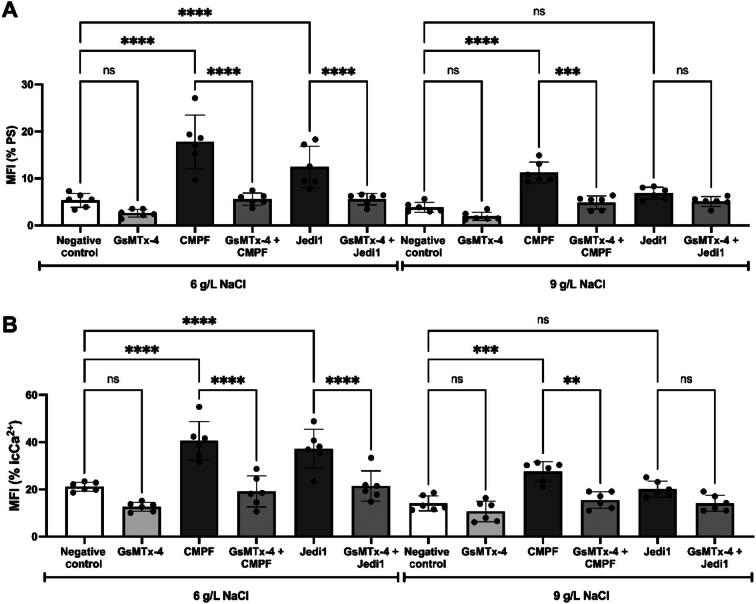
RBC PS exposure and icCa^2+^ in response to chemical stimulation. RBCs obtained from healthy subjects were incubated with or without 87 μM CMPF or Jedi1 for 30 min at room temperature and then incubated for 5 min with different NaCl concentrations (6 and 9 g/L). Negative control consisted of PBS with 4% HSA and 0.12% DMSO. RBCs were analyzed by flow cytometry of (**A**) annexin-V conjugated to APC to assess PS expression or (**B**) Fluo-4AM to measure intracellular calcium (icCa^2+^). RBCs were incubated with GsMTx-4 (2 µM) for 5 min before the chemical stimuli were applied. Results are expressed as MFI. **P < 0.01; ***P < 0.001; ****P < 0.0001 for the comparison of negative control versus CMPF or Jedi1; ns, non-significant for the comparison of negative control versus GsMTx-4 only.

By way of example, the PS exposure and icCa^2+^ histograms show the results from one blood donor (Supplementary Data Fig. S2). The aggregated results for eryptosis markers, PS and icCa^2+^, are shown in Fig. 3. Increased PS exposure was observed in RBCs following incubation with 87 µM of CMPF or Jedi1 (Fig. 3A) in 6 g/L NaCl (CMPF, 17.8 ± 5.7 MFI; Jedi1, 12.4 ± 4.4 MFI); negative control (5.3 ± 1.4 MFI) and incubations with GsMTx-4 alone (2.4 ± 0.8 MFI) showed no effect. Notably, at 9 g/L NaCl, CMPF—but not Jedi-1—showed increased PS exposure (CMPF, 13.2 ± 3.3 MFI; negative control 3.8 ± 1.1 MFI; Fig. 3A). In the presence of GsMTx-4, both CMPF and Jedi1 lost their ability to induce PS exposure (Fig. 3B). In the presence of GsMTx-4 in 6 g/L NaCl, the PS exposure induced by CMPF decreased to 5.5 ± 1.2 MFI; the Jedi1 effect decreased to 5.6 ± 1.2 MFI. In 9 g/L NaCl, the effect of CMPF was inhibited by GsMTx-4 (13.2 ± 3.3 versus 4.8 ± 1.3 MFI; Fig. 3B). No alterations were observed in incubations of RBC with GsMTx-4 alone (1.9 ± 0.8 MFI; Fig. 3B).

icCa^2+^ increased following incubation with 87 µM CMPF or Jedi1, but not with 2 µM of GsMTx-4 alone in 6 g/L NaCl (CMPF, 40.5 ± 8.1 MFI; Jedi1, 37.2 ± 8.2 MFI; GsMTx-4, 12.7 ± 1.9 MFI; negative control, 21.1 ± 1.8 MFI; Fig. 3B). Significantly increased icCa^2+^ levels were also observed following incubation with CMPF in 9 g/L NaCl (CMPF, 24.2 ± 4.7 MFI; negative control, 14.1 ± 3.1 MFI). The increase of icCa^2+^ was not significant with Jedi1 incubation and GsMTx-4 alone at 9 g/L NaCl (Jedi1, 21.8 ± 5.2 MFI; GsMTx-4, 10.7 ± 4.3 MFI; control, 14.1 ± 3.1 MFI) (Fig. 3B). GsMTx-4 blunted the icCa^2+^ increase with CMPF in 6 g/L NaCl (from 40.5 ± 8.1 to 19.1 ± 6.5 MFI); the same held true for incubation with Jedi1 (from 37.2 ± 8.2 to 21.4 ± 6.3 MFI; 3B). The CMPF effect in 9 g/L NaCl was blunted by GsMTx-4 (from 24.2 ± 4.7 to 15.4 ± 3.4 MFI; Fig. 3B).

## DISCUSSION

Our findings indicate that CMPF, at concentrations observed in dialysis patients, induces eryptosis, presumably through potential activation of PIEZO1 located on RBCs.

This research was motivated by several strands of evidence. First, in most patients with advanced CKD, the lifespan of RBCs is shortened and may thus contribute to the patient's anemia [29]. The premature RBC death is mostly due to eryptosis, a process where increased levels of icCa^2+^ drive a cascade of biochemical events that eventually result in phagocytosis of RBCs by macrophages [30]. Several studies have associated elevated eryptosis rates with uremic toxins [4, 5]. Second, stimulation of RBC PIEZO1 increases Ca^2+^ influx and elicits a series of intracellular events that change the cell's volume [31]. Third, while the physiological stimuli for PIEZO1 are mechanical in nature, at least four synthetic chemical PIEZO1 activators have been identified, two of which, Jedi1 and Jedi2, share striking structural similarities with the uremic solute CMPF. In healthy subjects, fasting CMPF levels are around 20–40 µM [32]. In uremia, CMPF concentrations up to 370 µM have been reported [33]. Lastly, several gain-of-function mutations in the PIEZO1 gene result in delayed PIEZO1 channel inactivation and prolonged Ca^2+^ influx, giving rise to dehydrated hereditary stomatocytosis, a hemolytic disorder [34].

On these grounds, we hypothesized that elevated CMPF may act as an endogenous chemical PIEZO1 activator that may trigger mechanisms that result in eryptosis [19]. Surface resonance binding assays revealed that Jedi1 acts through binding to the extracellular regions of the peripheral blade of PIEZO1 [16, 35]. Wang *et al*. demonstrated the importance of the 3-carboxylic acid methyl furan domain in PIEZO1 activation since the compound *N*-butylaniline, which lacks this domain, failed to bind to and activate the channel [16]. Of note, this 3-carboxylic acid methyl furan domain is shared by Jedi1 and CMPF [19] (Fig. 1). In the context of our research, it is important to note that Lohia *et al*. observed slightly increased hemolysis when RBCs were incubated with Jedi1 (100 µM, 300 µM, and 1 mM) for 30 min and then exposed to hypotonic stress [36]. Taken together, our results support the hypothesis that CMPF at concentrations observed in advanced CKD stages may prolong PIEZO1 activation in a Jedi1/Jedi2-like fashion and induce structural and functional alterations impairing RBC viability. However, a detailed understanding of the interaction between CMPF and PIEZO1 warrants further investigations of the entire PIEZO1 activation pathway.

Our study has some limitations. First, studies of RBCs from patients with CKD are desirable. It is important to note that such RBCs may already show increased icCa^2+^ levels and increased osmotic fragility [37, 38]. Second, we only explored some facets of the PIEZO1 activation pathways. Measuring icCa^2+^ concentration and PS exposure following a challenge to CMPF in a time- and dose-response manner will shed light on the interaction between CMPF and PIEZO1.

In summary, our results raise the possibility that CMPF at concentrations seen in uremia may induce eryptosis, a process also potentially influenced by other uremic toxins. The exact nature of the interaction between CMPF and PIEZO1 warrants future studies. If confirmed by other groups and complementary methods (e.g., patch clamping), our findings may open new therapeutic approaches to mitigate or even prevent premature death of RBCs in patients with advanced CKD.

## Supplementary Material

gfae275_Supplemental_File

## Data Availability

The experimental data are available from the corresponding author, Dr Peter Kotanko, upon reasonable request.
