# Supplementary material for: The uremic solute 3-carboxy-4-methyl-5-propyl-2-furanpropionate (CMPF) may enhance eryptosis and increase erythrocyte osmotic fragility through potential activation of PIEZO1
Source: Nephrol Dial Transplant. 2024 Nov 20;40(7):1342–9. doi: 10.1093/ndt/gfae275 (PMC12215662; doi:10.1093/ndt/gfae275)
Supplement: gfae275_Supplemental_File [file gfae275_Supplemental_File.docx]

**Supplementary Figures**

**Supplementary Figure S1**. Flow Cytometric Analysis of Isolated RBC Exposed to 0.12% DMSO vehicle in 6 g/L or 9 g/L Saline Solutions ………………………………………………..…………1

**Supplementary Figure S2**. Histograms for phosphatidylserine (PS) exposure and intracellular calcium (icCa^2+^) ………………………………………………………………………..…………2

**Supplementary Figure S1**. Flow Cytometric Analysis of Isolated RBC Exposed to 0.12% DMSO vehicle in 6 g/L or 9 g/L Saline Solutions.


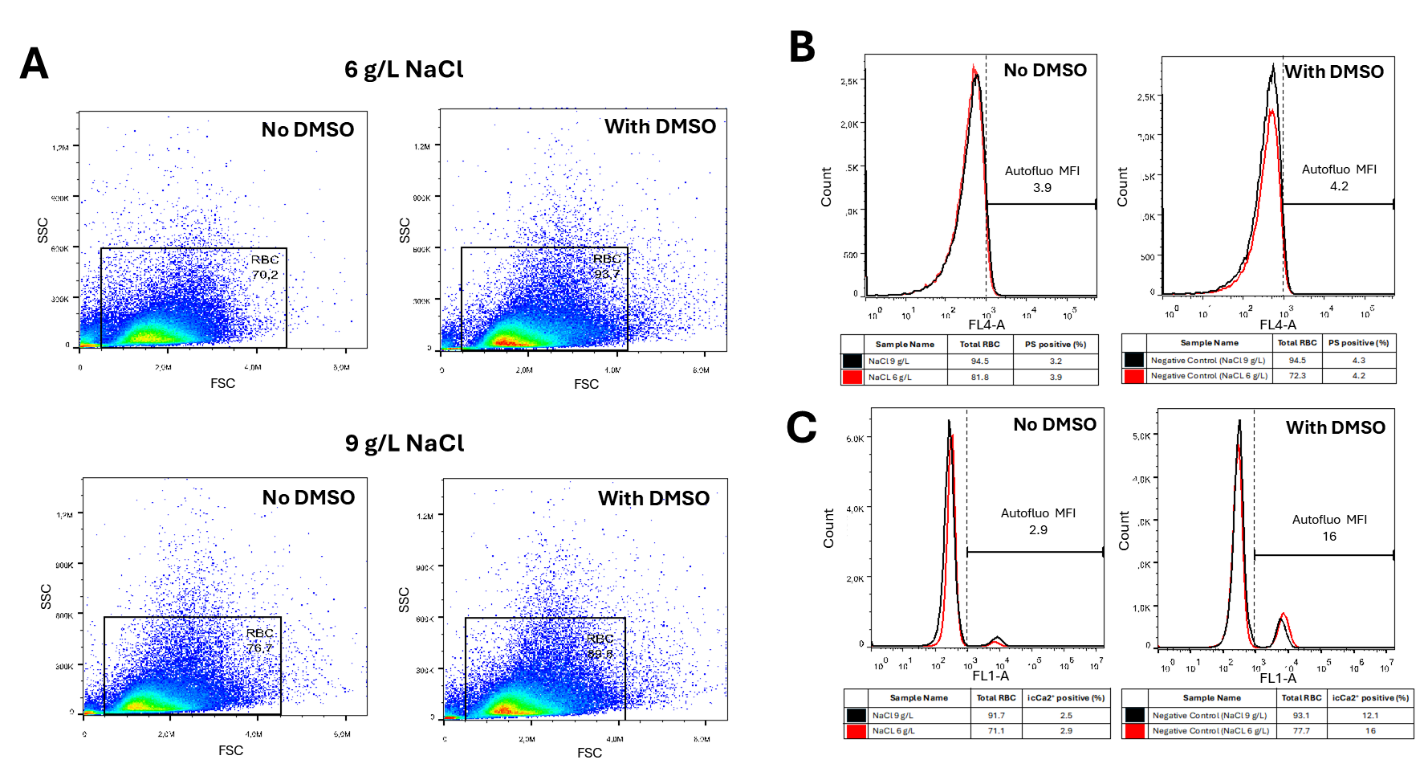


Panel A: Forward scatter (FSC) and side scatter (SSC) plots of isolated RBC. Cells were incubated in saline solutions of 6 g/L (top) and 9 g/L (bottom), either without (left) or with (right) 0.12% DMSO vehicle. Gates, indicated by black rectangles, were drawn in the FSC/SSC plots to select cells for further analysis. Panel B: Autofluorescence cutoff (dashed vertical line) for gated RBC incubated in 6 g/L NaCl (red histograms) or 9 g/L NaCl (black histograms), either without (left) or with (right) 0.12% DMSO vehicle, was determined using the FL4-A filter to quantitate mean fluorescence intensity (Autofluo MFI). Panel C: Autofluorescence cutoff was similarly determined using the FL1-A filter (Autofluo MFI).

**Supplementary Figure S2.** Histograms for phosphatidylserine (PS) exposure and intracellular calcium (icCa^2+^).


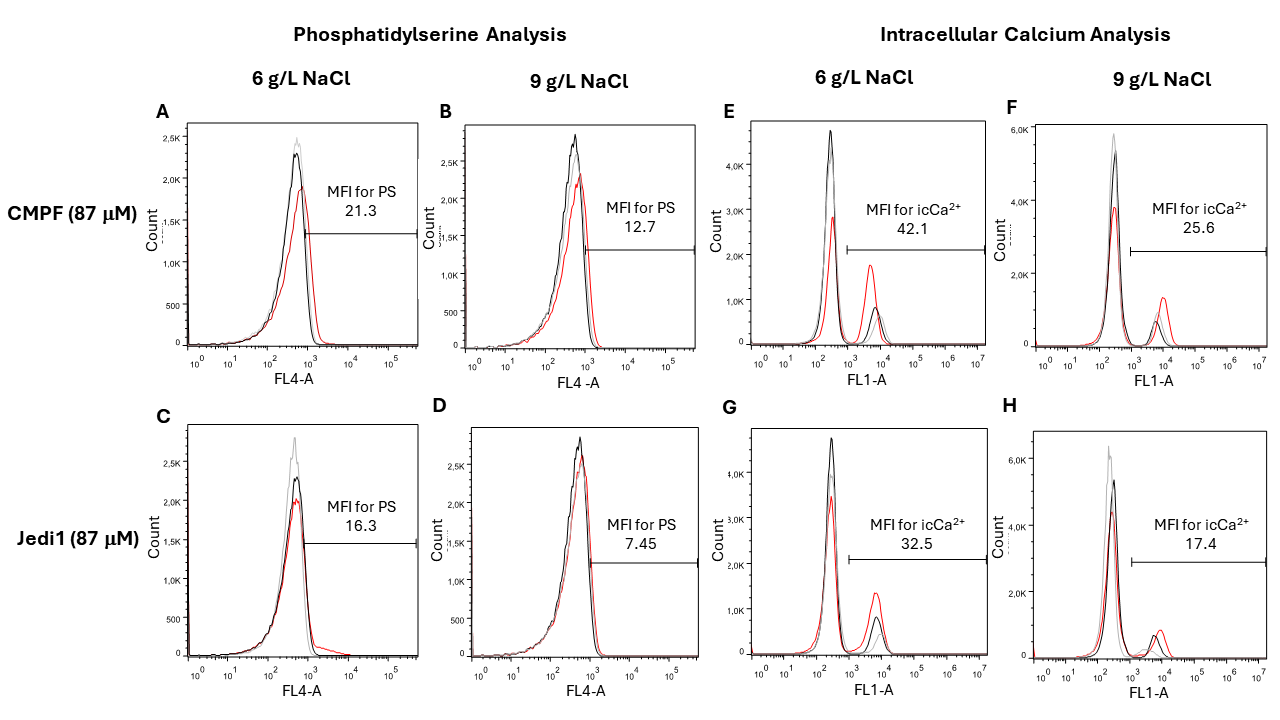


Representative Histograms - RBC preincubated or not with GsMTx-4 (Gray), and then incubated in Phosphate-Buffered Saline with 4 % Human Serum Albumin and 0.12% DMSO (Negative Control; black), CMPF (red) (A-B and E-F), or Jedi1 (red) (C-D and G-H), followed by incubation with 6 g/L NaCl (A, C and E, G) or 9 g/L NaCl (B, D and F, H). Mean Fluorescence Intensity (MFI) was analyzed by flow cytometry through Annexin-V-APC binding to Phosphatidylserine (MFI for PS) using FL4-A filter (A-D) or Fluo-4AM to measure intracellular calcium (MFI for icCa2+) using FL1-A filter (E-H).
